# Sex Differences in Cardiovascular-Kidney-Metabolic Syndrome: 30-Year US Trends and Mortality Risks—Brief Report

**DOI:** 10.1161/ATVBAHA.124.321629

**Published:** 2024-12-12

**Authors:** Hongwei Ji, Charumathi Sabanayagam, Kunihiro Matsushita, Ching-Yu Cheng, Tyler Hyungtaek Rim, Bin Sheng, Huating Li, Yih-Chung Tham, Susan Cheng, Tien-Yin Wong

**Affiliations:** 1 Tsinghua Medicine, Tsinghua University, Beijing, China (H.J., T.Y.W.).; 2 Department of Internal Medicine, Beijing Tsinghua Changgung Hospital, Beijing, China (H.J.).; 3 Singapore Eye Research Institute, Singapore National Eye Center (C.S., C.Y.C., T.H.R., Y.C.T., T.Y.W.).; 9 Department of Ophthalmology and Centre for Innovation and Precision Eye Health, Yong Loo Lin School of Medicine, National University of Singapore (Y.C.T.).; 4 Ophthalmology and Visual Science Academic Clinical Program, Duke-NUS Medical School, Singapore (C.S., C.Y.C., Y.C.T.).; 5 Department of Epidemiology, Johns Hopkins Bloomberg School of Public Health, Baltimore, MD (K.M.).; 6 Department of Computer Science and Engineering, Shanghai Jiao Tong University, China (B.S.).; 7 Department of Endocrinology and Metabolism, Shanghai Diabetes Institute, Shanghai Clinical Center for Diabetes, Shanghai Key Laboratory of Diabetes Mellitus, Shanghai Sixth People’s Hospital Affiliated to Shanghai Jiao Tong University School of Medicine, China (H.L.).; 8 Department of Cardiology, Smidt Heart Institute, Cedars-Sinai Medical Center, Los Angeles, CA (S.C.).

**Keywords:** cardiovascular diseases, kidney diseases, metabolic diseases, obesity, sex characteristics

## Abstract

**BACKGROUND:**

The American Heart Association recently published guidelines on how to clinically identify and categorize individuals with cardiovascular-kidney-metabolic (CKM) syndrome. The extent to which CKM syndrome prevalence and prognosis differ by sex remains unknown. This study aimed to examine the impact of sex on trends in prevalence over 30 years and the long-term prognosis of CKM syndrome in the United States.

**METHODS:**

We analyzed nationally representative National Health and Nutrition Examination Survey 1988 to 2018 data collected from 33 868 US adults (aged ≥20 years) who were under surveillance for all-cause mortality through December 31, 2019. We examined the sex-specific prevalence of CKM syndrome and sex-specific CKM associations with all-cause mortality.

**RESULTS:**

Of the 33 868 adults studied, the mean±SD age was 48.4±18.3 years with 52% women and 56% non-White. Overall prevalence of CKM syndrome increased steadily from 1988 to 2018 in both sexes, with a larger temporal rise in prevalent stage 3 CKM seen for men (from 18.9% to 22.4%) compared with women (from 13.9% to 15.2%). Over a median follow-up of 13.3 years, there were 8745 deaths. In the multivariable Cox regression analysis, worsening CKM severity was associated with all-cause mortality (*P*<0.001 for both sexes), with greater magnitudes of risk seen in women (hazards ratio, 1.24–3.33) compared with men (hazards ratio, 0.85–2.60) across all stages (likelihood ratio test χ^2^, 19.0; *P*_interaction_<0.001); results were similar for cardiovascular mortality (likelihood ratio test χ^2^, 22.3; *P*_interaction_<0.001).

**CONCLUSIONS:**

Women, compared with men, exhibited a lower prevalence of CKM stage 3 but experienced excess mortality risk across the spectrum of multisystem CKM dysfunction. These findings underscore the importance of identifying mechanisms underlying joint cardiovascular, kidney, and metabolic system pathophysiology to close a potentially widening sex disparities gap in multiorgan disease risk.

**Highlights**

- The overall prevalence of cardiovascular-kidney-metabolic syndrome increased steadily from 1988 to 2018 in both sexes, with a larger rise seen for prevalent stage 3 cardiovascular-kidney-metabolic among men (from 18.9% to 22.4%) compared with women (from 13.9% to 15.2%).
- Multivariable-adjusted hazards for all-cause mortality increased with worsening cardiovascular-kidney-metabolic severity, with greater magnitudes of risk seen in women compared with men across all stages.
- We similarly observed greater magnitudes of cardiovascular mortality risk in women than in men across cardiovascular-kidney-metabolic stages.

Given the rapidly growing appreciation for the pathophysiological interplay between cardiometabolic risk factors, chronic kidney disease (CKD), and cardiovascular disease (CVD), the American Heart Association recently published expert recommendations on how to clinically identify and categorize individuals with cardiovascular-kidney-metabolic (CKM) syndrome.^1^ This framework has paved the way for discerning new insights about the interconnected and aggregate risks experienced by individuals with clustering of CKM traits.^2^ Currently, there are gaps in our knowledge due to an incomplete understanding of how prevalence and prognosis of CKM may differ by sex,^1^ despite previously recognized sex differences about manifestations and outcomes associated with each of the individual CKM traits.^3,4^ In particular, women consistently demonstrate greater CVD and mortality risks than men in the setting of obesity and diabetes.^3^ Similarly, women compared with men with kidney disease have variably greater adjusted mortality risk, especially in the setting of kidney failure and younger age.^4,5^ Thus, the confluence of preclinical and clinical CKM traits across earlier and later stages of coexisting disease may reveal even more pronounced sex differences than previously observed. Our aim is to investigate potential sex differences in the prevalence and prognosis of CKM syndrome.

## Methods

All data and materials have been made publicly available by the Centers for Disease Control and Prevention and can be accessed at https://www.cdc.gov/nchs/nhanes/index.htm.

We analyzed National Health and Nutrition Examination Survey (NHANES; 1988–2018) data that were collected from nationally representative US population samples over the past 30 years. Each study participant completed a personal interview and underwent a physical examination at the mobile examination center.^6^ Clinical covariates and serum biomarkers were assessed as previously reported.^6^ Participants’ identifiers were linked to the National Death Index through December 31, 2019. Of all 132 627 participants in NHANES 1988 to 2018 examinations (Figure S1), we excluded individuals who were aged <20 years (n=60 516) or had incomplete information on key covariates or outcomes (n=38 243).

For the final sample of 33 868 adults, we defined stages of CKM syndrome according to criteria provided in the American Heart Association scientific statement with adaptations to accommodate for the type and availability of data in NHANES. Specifically, CKM syndrome stages were defined as previously detailed^1^: stage 0 (normal body mass index and waist circumference, normoglycemia, normotension, a normal lipid profile, and no evidence of CKD or subclinical or clinical CVD); stage 1 (overweight/obesity [body mass index ≥25 kg/m^2^], abdominal obesity [waist circumference ≥88 cm for women or 102 cm for men], or prediabetes [fasting blood glucose ≥100–125 mg/dL or HbA1c between 5.7% and 6.4%], without the presence of other metabolic risk factors or CKD); stage 2 (presence of metabolic risk factors [triglycerides ≥135 mg/dL, hypertension, metabolic syndrome, and diabetes] or CKD); stage 3 (risk equivalents of subclinical CVD including very high-risk CKD [stage G4 or G5 CKD or very high-risk per Kidney Disease: Improving Global Outcomes classification] or 10-year atherosclerotic CVD risk ≥10%); and stage 4 (coronary heart disease, heart failure, or stroke among individuals with excess/dysfunctional adiposity, other CKM risk factors, or CKD). Metabolic syndrome was defined as previously detailed: ≥3 of the following: waist circumference ≥88 cm for women and ≥102 cm for men; high-density lipoprotein cholesterol <40 mg/dL for men or <50 mg/dL for men; triglycerides ≥150 mg/dL; systolic blood pressure ≥130 mm Hg or diastolic blood pressure ≥80 mm Hg or use of antihypertensive medications; and fasting blood glucose ≥100 mg/dL.

We estimated the sex-stratified population prevalence of CKM stages using sampling weights to account for the complex, multistage sample design of NHANES (Table S1). After confirming the proportionality assumption, we then used sex-specific Cox proportional hazards analyses to examine CKM stages in relation to all-cause mortality adjusting for factors not included in CKM definitions: age, race, current smoking, family income, and education level. We used the χ^2^ test to compare the prevalence of CKM syndrome stages by sex. Next, we included multiplicative terms to assess for interaction between sex and CKM syndrome stage in the Cox proportional hazards regression models and, in turn, evaluate whether relative hazards significantly differed between women and men across CKM stages. We also tested for overall interactions between sex and CKM stages using the likelihood ratio test in comparisons of models with and without the terms for interaction between sex and CKM syndrome stages. In sensitivity analyses, we performed multiple imputations by chained equations involving an iterative series of predictive mean matching, logistic regression, and polytomous regression models to impute data on key variables that were missing from the original source data set.^7^ We then repeated our main analyses on the larger complete data set that included imputed values and compared results with those of the primary analyses. All analyses were conducted using R, v4.2.1, and a 2-tailed *P*<0.05 was considered statistically significant.

## Results

The demographics and clinical characteristics of the study sample are shown in Table S2. Of the 33 868 US adults studied, the mean±SD age was 48.4±18.3 years, and there were 51.7% women and 55.6% individuals of non-White race. Women compared with men had more favorable cardiometabolic profiles, less prevalent CKD, and lower atherosclerotic CVD risk scores (*P*≤0.005 for all). The overall prevalence of CKM syndrome increased steadily from 1988 to 2018 in both sexes, with a larger rise seen for prevalent stage 3 CKM among men (from 18.9% to 22.4%) compared with women (from 13.9% to 15.2%; Figure).

**Figure.**
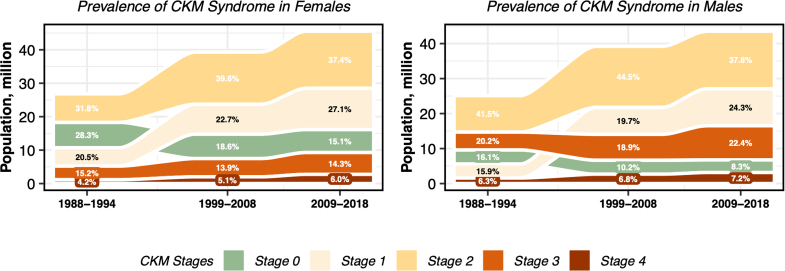
**Thirty-year prevalence of cardiovascular-kidney-metabolic (CKM) syndrome by sex.** As shown, the overall prevalence of at least stage 1 CKM increased steadily over time, particularly among men with respect to the prevalence of stage 3 CKM.

Over a median follow-up of 13.3 years, there were a total of 8745 deaths with higher crude mortality rates seen across advancing stages of CKM (*P*<0.001). As shown in the Table, the unadjusted mortality rates were higher in women from CKM stage 2 to stage 3 but higher in men with stage 4 CKM. In Cox regression models, multivariable-adjusted hazards for all-cause mortality increased with worsening CKM severity (*P*<0.001), with greater magnitudes of risk seen in women compared with men across all stages (likelihood ratio test χ^2^=19.0; *P*<0.001 for sex interaction). We similarly observed greater magnitudes of cardiovascular mortality risk in women than in men (likelihood ratio test χ^2^=22.3; *P*<0.001 for sex interaction) across CKM stages (Table S3).

**Table.**
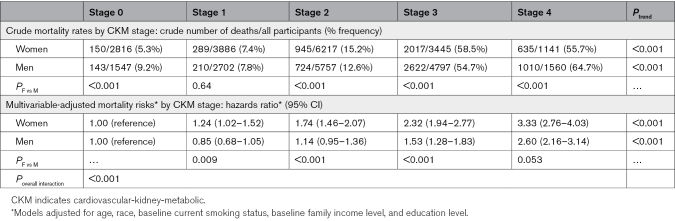
Sex-Specific Association of CKM Syndrome With All-Cause Mortality

Because over half of the source cohort of participants were excluded due to missing data for key variables (Table S4), we conducted sensitivity analyses using a data set with these originally missing values imputed. Specifically, we used multiple imputations by chained equations (involving 5 iterations of modeling) to generate a data set of n=72 111 with nonmissing values. Analyzing this larger data set generated findings that were similar to those in the primary analyses including a magnitude of mortality risk that was more evident in women than in men (*P*<0.001 to *P*=0.013 for sex interactions; Table S5).

## Discussion

In this representative US population cohort study spanning over 30 years, we found that while the prevalence of CKM syndrome has increased steadily over time in both sexes, the associated mortality risks have been significantly higher in women than men.^8^ Given the focus on sex-specific data collected over 3 decades, our study extends from recent reports of how the CKD syndrome and its component overlapping cardiac, renal, and metabolic conditions have become increasingly common across adult populations in the United States^8,9^

With respect to classically defined cardiometabolic risk traits, prior studies have shown how prevalence has increased over time and similarly in both sexes.^10^ The CKM syndrome more broadly defines metabolic risk, and in this context, the prevalence of particularly the advanced CKM stages has been rising more prominently in men than in women. This trend aligns with the consistent observation that men accumulate more visceral and liver fat than women, both of which increase the risk for diabetes and CKD.^11–14^ However, despite men developing a greater prevalence of more severe CKM risk burden, we found that women exhibited proportionately greater CKM-related CVD risk. Prior studies have consistently reported that women experience greater CVD risk than men in the setting of classic metabolic traits such as obesity, insulin resistance, and type 2 diabetes.^11,15–18^ Although underlying mechanisms remain unclear, these findings may be related to the women cardiovascular system, particularly the vasculature, being more sensitive to similar dose exposures of metabolic stress. Accordingly, data from multiple studies now indicate that trajectories of age-related vascular dysfunction are more pronounced in women, especially in the setting of metabolic risk burden.^18,19^ In fact, the excess vascular response to metabolic stress in women has been seen in the setting of not only obesity and diabetes but also steatotic liver disease.^18,20,21^ Interestingly, with respect to CKD, prior studies indicate that prevalence is greater in women,^22,23^ and yet, attributable CVD risks are greater in men before renal failure. Among individuals with more severe CKD and overt renal failure, however, nonfatal CVD event risks seem greater in women^24–27^; in addition, women still exhibit greater vascular sensitivity than men across the spectrum of CKD risk even as early as during prepubertal age ranges.^28^ Taken together, the broader CKM definitions seem to further highlight the excess CVD risks that women have previously demonstrated in relation to metabolic disease burden and more severe CKD, potentially due to differential vascular responses to metabolic stress in addition to the previously described effects of sex hormones, sex chromosomes, and variable sex-dependent access to risk modifying treatments.^29,30^ Additional potential contributors include sex variation in epigenetic and direct metabolic responses to life course stressors, including, but not limited to, nutritional intake patterns (eg, sex-specific palatability and ingestion of highly processed foods), psychosocial stressors, and behavioral and lifestyle factors.^31–36^

Several limitations of our study should be considered. We relied on a single national cohort and needed to adapt the American Heart Association definitions of CKM stages to accommodate the variables available in NHANES, as others have done.^8^ Accordingly, a large proportion of the NHANES cohort had missing data on key variables for determining CKM status; fortunately, sensitivity analyses conducted on rigorously imputed data revealed consistent results. Notwithstanding the need for additional separate cohort studies to validate our results and determine broader generalizability, the study findings are derived from a large series of cross-sectional NHANES survey data collected from individuals representative of the US population.

Our overall study results indicate that the co-occurrence of CKM traits, even in the earliest stages of subclinical disease progression, is a marker of elevated risk, especially in women compared with men. Given that prior studies have indicated excess adverse vascular responses to metabolic stress in women, the current results suggest that multisystem CKM dysfunction may serve to effectively contribute to and even augment these effects. Additional investigations are needed to identify underlying mechanisms and evaluate the ability of targeted and potentially sex-specific interventions to mitigate risks, at least some of which may include vascular and metabolic therapies.^37,38^ Additional studies may also clarify how sex differences may be integrated into the clinical evaluation of CKM risks, including in the early stages of CKM syndrome, as part of approaches to closing a sex disparities gap that, with growing prevalence, may well be widening over time.

## ARTICLE INFORMATION

### Acknowledgments

H. Ji had full access to all the data in the study and took responsibility for the integrity of the data and the accuracy of the data analysis. H. Ji was involved in the acquisition of data. All authors were involved in the analysis and interpretation of data. All authors were involved in the drafting of the article. All authors were involved in critical revision of the article for important intellectual content. H. Ji, S. Cheng, and T. Yin Wong obtained funding. S. Cheng and T. Yin Wong were involved in study supervision.

### Sources of Funding

This study was funded in part by the National Key Research and Development Program of China (grant 2022YFC2502800), the National Natural Science Foundation of China (grants 82388101 and 82103908), the China Postdoctoral Science Foundation (grant 2024M751733), the Beijing Natural Science Foundation (grant IS23096), the Shandong Provincial Natural Science Foundation (grant ZR2021QH014), the Shuimu Scholar Program of Tsinghua University (grant 2023SM196), the National Postdoctoral Innovative Talent Support Program (grant BX20230189), the Erika J Glazer Family Foundation, and the National Institutes of Health grant U54AG065141. The funding sources had no role in the design and conduct of the study; collection, management, analysis, and interpretation of the data; preparation, review, or approval of the article; and decision to submit the article for publication.

### Disclosures

None.

### Supplemental Material

Tables S1–S5

Figure S1

Major Resources Table

## Supplementary Material
