# Supplementary material for: Sex Differences in Cardiovascular-Kidney-Metabolic Syndrome: 30-Year US Trends and Mortality Risks—Brief Report
Source: Arterioscler Thromb Vasc Biol. 2024 Dec 12;45(1):157–61. doi: 10.1161/ATVBAHA.124.321629 (PMC11729504; doi:10.1161/ATVBAHA.124.321629)
Supplement: Supplementary file 1 [file atv-45-157-s001.pdf]

SUPPLEMENTAL MATERIALS

Title: Sex Differences in Cardiovascular-Kidney-Metabolic Syndrome: 30-Year U.S. Trends and Mortality Risks

Hongwei Ji[1,2], Charumathi Sabanayagam[3,4], Kunihiro Matsushita[5], Ching Yu Cheng[3,4], Tyler Hyungtaek Rim[3], Bin Sheng[6], Huating Li[7], Yih Chung Tham[3,4], Susan Cheng[8#], Tien Yin Wong[1,3#]

1. Tsinghua Medicine, Tsinghua University, Beijing, China
2. Department of Internal Medicine, Beijing Tsinghua Changgung Hospital, Beijing,
3. Singapore Eye Research Institute, Singapore National Eye Center, Singapore, Singapore
4. Ophthalmology and Visual Science Academic Clinical Program, Duke-NUS Medical School, Singapore, Singapore.
5. Department of Epidemiology, Johns Hopkins Bloomberg School of Public Health, Baltimore, MD, USA
6. Department of Computer Science and Engineering, Shanghai Jiao Tong University, Shanghai, China
7. Department of Endocrinology and Metabolism, Shanghai Diabetes Institute, Shanghai Clinical Center for Diabetes, Shanghai Key Laboratory of Diabetes Mellitus, Shanghai Sixth People's Hospital Affiliated to Shanghai Jiao Tong University School of Medicine, Shanghai, China
8. Department of Cardiology, Smidt Heart Institute, Cedars-Sinai Medical Center, Los Angeles, CA, USA

#Senior Authors

Correspondence:
Prof. Susan Cheng, Department of Cardiology, Smidt Heart Institute, Cedars-Sinai Medical Center, Los Angeles, CA, USA Email Susan.Cheng@cshs.org;
Prof. Tien Yin Wong, Tsinghua Medicine, Tsinghua University, Beijing, 100084, China Email ophwty@nus.edu.sg;

Figure S1. Sampling Strategy. Note that CKM definitions relied on a pre-specified set of demographic, anthropometric, and laboratory measures and variables. Data from 33,868 adult participants collected over 30 years (1988-2018) were included in final analyses.

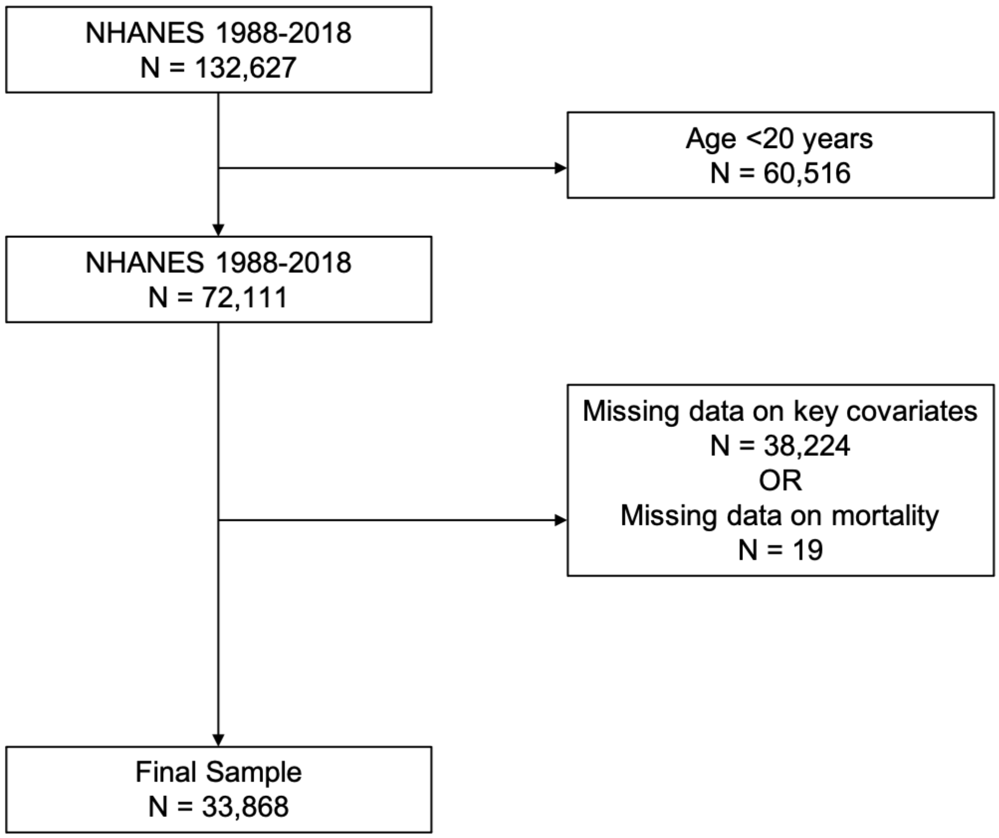

Table S1. Number of Participants in Each of the NHANES Survey Cycles with Weight Adjustments.

	National Health and Nutrition Examination Survey Cycles										
	**1988-1994**	**1999-2000**	**2001-2002**	**2003-2004**	**2005-2006**	**2007-2008**	**2009-2010**	**2011-2012**	**2013-2014**	**2015-2016**	**2017-2018**
No. of Participants	14424	1599	1929	1703	1796	2105	2284	2053	2180	1909	1886
Weight Adjustments*	wt/3	wt/5*2	wt/5*2	wt/5	wt/5	wt/5	wt/5	wt/5	wt/5	wt/5	wt/5

*To allow for each of study periods spanning across decades comparable, as denoted in differently colored background shades, we applied weight adjustments according to previously established methods as outlined in: https://wwwn.cdc.gov/nchs/nhanes/tutorials/weighting.aspx,

Table S2. Baseline Characteristics of Participants by Sex

	Overall	*Females*	*Males*	*P value**
N	33868	17505	16363	
Age, mean (SD), year	48.4 (18.3)	48.0 (18.4)	49.0 (18.3)	<0.001
Race, n (%)				0.005
African American	7575 (22.4)	4038 (23.1)	3537 (21.6)	
White	11242 (33.2)	7724 (44.1)	7327 (44.8)	
Other	15051 (44.4)	5743 (32.8)	5499 (33.6)	
Systolic BP, mean (SD), mmHg	123.52 (19.73)	121.50 (21.10)	125.68 (17.91)	<0.001
Diastolic BP, mean (SD), mmHg	71.07 (12.88)	69.03 (12.50)	73.24 (12.93)	<0.001
Antihypertensives, n (%)	8410 (24.8)	4509 (25.8)	3901 (23.8)	<0.001
BMI, mean (SD), kg/m^2	28.10 (6.24)	28.47 (6.91)	27.69 (5.42)	<0.001
Current Smoking, n (%)	8246 (24.3)	3568 (20.4)	4678 (28.6)	<0.001
DM, n (%)	3199 (9.4)	1550 (8.9)	1649 (10.1)	<0.001
HDL, mean (SD), mg/dL	52.53 (15.89)	56.93 (16.21)	47.83 (14.09)	<0.001
LDL, mean (SD), mg/dL	118.79 (36.85)	118.19 (37.13)	119.43 (36.54)	0.008
TC, mean (SD), mg/dL	199.73 (43.37)	202.39 (44.27)	196.89 (42.20)	<0.001
CKD stage, n (%)				<0.001
Normal	27760 (82.0)	14195 (81.1)	13565 (82.9)	
G1	2095 (6.2)	1224 (7.0)	871 (5.3)	
G2	1569 (4.6)	779 (4.5)	790 (4.8)	
G3a	1644 (4.9)	879 (5.0)	765 (4.7)	
G3b	595 (1.8)	316 (1.8)	279 (1.7)	
G4	148 (0.4)	88 (0.5)	60 (0.4)	
G5	57 (0.2)	24 (0.1)	33 (0.2)	
10-yr ASCVD Risk, mean (SD), %	9.70 (13.11)	7.98 (12.10)	11.55 (13.88)	<0.001
Clinical CVD, n (%)	2708 (8.0)	1143 (6.5)	1565 (9.6)	<0.001
CKM Stages, n (%)				<0.001
Stage0	4363 (12.9)	2816 (16.1)	1547 (9.5)	
Stage1	6588 (19.5)	3886 (22.2)	2702 (16.5)	
Stage2	11974 (35.4)	6217 (35.5)	5757 (35.2)	
Stage3	8242 (24.3)	3445 (19.7)	4797 (29.3)	
Stage4	2701 (8.0)	1141 (6.5)	1560 (9.5)	

Abbreviations: SD: standard deviation, BP: blood pressure, BMI: body mass index, DM: diabetes mellitus, HDL: high-density lipoprotein cholesterol, LDL: low-density lipoprotein cholesterol, TC:

total cholesterol, CKD: chronic kidney disease, ASCVD: atherosclerotic cardiovascular disease risk score, CVD: cardiovascular disease, CKM: cardiovascular-kidney-metabolic syndrome. CVD includes coronary heart disease, heart failure, or stroke. * P values show are calculated for between-sex differences, using t-test and chi-square test, as appropriate.

Table S3. Sex-Specific Associations of Cardiovascular-Kidney-Metabolic Syndrome with Cardiovascular Mortality.

	**Crude Cardiovascular Mortality Rates, by CKM Stage** *Crude No. of Cardiovascular Deaths / All Participants (% Frequency)*					
	**Stage 0**	**Stage 1**	**Stage 2**	**Stage 3**	**Stage 4**	***P*** ***trend***
**Females**	24/2816 (0.9%)	57/3886 (1.5%)	254/6217 (4.1%)	775/3445 (22.5%)	285/1141 (25%)	<0.001
**Males**	23/1547 (1.5%)	46/ 2702 (1.7%)	186/5757 (3.2%)	879/4797 (18.3%)	503/1560 (32.2%)	<0.001
$P_{F\ vs.\ M}$	0.074	0.51	0.015	<0.001	<0.001	-

	**Multivariable-Adjusted Cardiovascular Mortality Risks, by CKM Stage** *Hazards Ratio* (95% CI)*					
	**Stage 0**	**Stage 1**	**Stage 2**	**Stage 3**	**Stage 4**	***P*** ***trend***
**Females**	1.00 [Reference]	1.49 (1.92, 2.41)	2.78 (1.82, 4.23)	4.80 (3.15, 7.34)	8.23 (5.35, 12.67)	<0.001
**Males**	1.00 [Reference]	1.11 (0.67, 1.83)	1.74 (1.13, 2.69)	2.71 (1.71, 4.16)	6.88 (4.46, 10.61)	<0.001
$P_{F\ vs.\ M}$	-	0.40	0.12	0.053	0.55	-
$P_{overall\ interaction}$			<0.001			

Abbreviations: CKM: cardiovascular-kidney-metabolic syndrome, CI: confidence interval.

Table S4. Missingness of Data for Key Variables in the Source Cohort.

Variable, Unit	Frequency of Missingness, n (%)
Systolic BP, mmHg	7324 (10.1)
Diastolic BP, mmHg	7324 (10.1)
BMI, kg/m^2	3812 (5.2)
Waist Circumference, cm	7017 (9.7)
Fasting Glucose, mg/dL	31,023 (43.0)
HbA1c, %	6134 (8.5)
Triglycerides, mg/dL	32,429 (44.9)
HDL, mg/dL	7019 (9.7)
Smoking, n	468 (0.6)
UACR, mg/g	4524 (6.2)
Poverty-to-Income Ratio	5317 (7.3)
Education Attainment	6 (0)
Death, n	148 (0.2)
Time to Death, years	3260 (4.5)
Congestive Heart Failure, n	3 (0)
Stroke, n	3 (0)
Myocardial Infarction, n	3 (0)

Abbreviations**:** BP: blood pressure, BMI: body mass index, HDL: high-density lipoprotein cholesterol, UACR: urinary albumin creatinine ratio.

Table S5. Multivariable-Adjusted Mortality Risks Estimated From Datasets with Originally Missing Data Imputed (N=72,111). In sensitivity analyses, we performed multiple imputation by chained equations (MICE) to impute data on key variables that were missing from the original source dataset. From an iterative series of predictive mean matching, logistic regression, and polytomous regression models, a total of 5 dataset versions were generated that were deemed to have convergent data features and, accordingly, results of repeating statistical analyses applied to each of these datasets (versions A through E, below) revealed consistently similar results.

**A.**	**Multivariable-Adjusted Mortality Risks, by CKM Stage** *Hazards Ratio* (95% CI)*					
	Stage 0	Stage 1	Stage 2	Stage 3	Stage 4	P_{trend}
**Females**	1.00 [Reference]	1.19 (1.00, 1.42)	1.66 (1.42, 1.93)	2.41 (2.06, 2.81)	3.91 (3.34, 4.58)	<0.001
**Males**	1.00 [Reference]	0.84 (0.70, 1.01)	1.11 (0.95, 1.30)	1.57 (1.35, 1.83)	2.64 (2.25, 3.10)	<0.001
$P_{F\ vs.\ M}$	-	0.006	<0.001	<0.001	<0.001	-
$P_{overall\ interaction}$			<0.001			

**B.**	**Multivariable-Adjusted Mortality Risks, by CKM Stage** *Hazards Ratio* (95% CI)*					
	Stage 0	Stage 1	Stage 2	Stage 3	Stage 4	P_{trend}
**Females**	1.00 [Reference]	1.19 (1.00, 1.41)	1.61 (1.39, 1.87)	2.36 (2.03, 2.75)	3.76 (3.22, 4.40)	<0.001
**Males**	1.00 [Reference]	0.88 (0.73, 1.06)	1.12 (0.96, 1.31)	1.62 (1.38, 1.89)	2.72 (2.32, 3.19)	<0.001
$P_{F\ vs.\ M}$	-	0.018	0.001	<0.001	0.002	-
$P_{overall\ interaction}$			0.013			

**C.**	**Multivariable-Adjusted Mortality Risks, by CKM Stage** *Hazards Ratio* (95% CI)*					
	Stage 0	Stage 1	Stage 2	Stage 3	Stage 4	P_{trend}
**Females**	1.00 [Reference]	1.21 (1.02, 1.44)	1.69 (1.45, 1.96)	2.42 (2.07, 2.82)	3.91 (3.34, 4.58)	<0.001
**Males**	1.00 [Reference]	0.85 (0.70, 1.02)	1.16 (0.99, 1.36)	1.61 (1.38, 1.89)	2.76 (2.35, 3.24)	<0.001
$P_{F\ vs.\ M}$	-	0.006	<0.001	<0.001	0.001	-
$P_{overall\ interaction}$			0.007			

**D.**	**Multivariable-Adjusted Mortality Risks, by CKM Stage** *Hazards Ratio* (95% CI)*					
	Stage 0	Stage 1	Stage 2	Stage 3	Stage 4	P_{trend}
**Females**	1.00 [Reference]	1.19 (1.00, 1.41)	1.61(1.39, 1.87)	2.38 (2.04, 2.77)	3.84 (3.29, 4.49)	<0.001
**Males**	1.00 [Reference]	0.85 (0.70, 1.02)	1.12 (0.96, 1.31)	1.59 (1.36, 1.86)	2.67 (2.27, 3.13)	<0.001
$P_{F\ vs.\ M}$	-	0.008	0.001	<0.001	<0.001	-
$P_{overall\ interaction}$			0.003			

**E.**	**Multivariable-Adjusted Mortality Risks, by CKM Stage** *Hazards Ratio* (95% CI)*					
	Stage 0	Stage 1	Stage 2	Stage 3	Stage 4	P_{trend}
**Females**	1.00 [Reference]	1.16 (0.97, 1.37)	1.62(1.40, 1.89)	2.40 (2.06, 2.79)	3.87 (3.32, 4.53)	<0.001
**Males**	1.00 [Reference]	0.92 (0.77 1.11)	1.16 (0.99, 1.36)	1.66 (1.42, 1.94)	2.86 (2.43, 3.36)	<0.001

$P_{F\ vs.\ M}$	-	0.078	0.002	<0.001	0.005	-
$P_{overall\ interaction}$			0.004			

***Abbreviations*: CKM: cardiovascular-kidney-metabolic syndrome. CI: confidence interval.

Major Resources Table

In order to allow validation and replication of experiments, all essential research materials listed in the Methods should be included in the Major Resources Table below. Authors are encouraged to use public repositories for protocols, data, code, and other materials and provide persistent identifiers and/or links to repositories when available. Authors may add or delete rows as needed.

Data & Code Availability

Description	Source / Repository	Persistent ID / URL
NHANES dataset	Centers for Disease Control and Prevention (CDC)	https://www.cdc.gov/nchs/nhanes/index.htm
